# Cortical Thickness Changes in Chronic Ketamine Users

**DOI:** 10.3389/fpsyt.2021.645471

**Published:** 2021-03-25

**Authors:** Jun Zhong, Huawang Wu, Fengchun Wu, Hongbo He, Zhaohua Zhang, Jiaxin Huang, Penghui Cao, Ni Fan

**Affiliations:** The Affiliated Brain Hospital of Guangzhou Medical University, Guangzhou Huiai Hospital, Guangzhou, China

**Keywords:** ketamine, ketamine dependent, frontal lobes, gray matter, cortical thickness, surface-based morphometry

## Abstract

**Background:** Previous studies have examined the effects of long-term ketamine use on gray matter volume. But it is unclear whether chronic ketamine use alters cortical thickness and whether cortical thickness changes in chronic ketamine users are associated with cognitive deficits observed in chronic ketamine users.

**Methods:** Here, 28 chronic ketamine users and 30 healthy controls (HCs) were recruited. Cortical morphometry based on Computational Anatomy Toolbox (CAT12) was used to measure cortical thickness. Cognitive performance was measured by MATRICS Consensus Cognitive Battery (MCCB). Two-sample *t*-test was used to assess differences in cortical thickness and cognitive performance between the two groups. Partial correlation analysis was used for assessing correlations between cortical thickness changes and clinical characteristics, cognitive performance in chronic ketamine users.

**Results:** Chronic ketamine users exhibited significantly reduced cortical thickness in frontal, parietal, temporal, and occipital lobes compared to HC [false discovery rate (FDR) corrected at *p* < 0.05]. In chronic ketamine users, the average quantity (g) of ketamine use/day was negatively correlated with cortical thickness in the left superior frontal gyrus (SFG), right caudal middle frontal gyrus (MFG), and right paracentral lobule. The frequency of ketamine use (days per week) was negatively correlated with cortical thickness in the left isthmus cingulate cortex. Duration of ketamine use (month) was negatively correlated with cortical thickness in the left precentral gyrus. The chronic ketamine users showed significantly poorer cognitive performance on the working memory (*P* = 0.009), visual learning (*P* = 0.009), speed of processing (*P* < 0.000), and Matrics composite (*P* = 0.01). There was no correlation between scores of domains of MCCB and reduced cortical thickness.

**Conclusion:** The present study observed reduced cortical thickness in multiple brain areas, especially in the prefrontal cortex (PFC) in chronic ketamine users. Dose, frequency, and duration of ketamine use was negatively correlated with cortical thickness of some brain areas. Our results suggest that chronic ketamine use may lead to a decrease of cortical thickness. But the present study did not observe any correlation between reduced cortical thickness and decreased cognitive performance in chronic ketamine users.

## Introduction

Ketamine, a derivative of phencyclidine, is a non-competitive antagonist of N-methyl-D-aspartate glutamate receptor (1). Over the past two decades, ketamine has become one of the most commonly abused drugs in mainland China (2–4). Concerns about the consequences of chronic ketamine use are increasing. Ketamine administration in rodents led to a dose- and time-dependent increase in neuronal cell death in the frontal cortex (5), hippocampus, and thalamus (5). It suggested that chronic ketamine use probably results in abnormal brain structure changes. A voxel-based morphometric (VBM) study found that the gray matter volume of the prefrontal cortex (PFC) decreased in chronic ketamine users, and these abnormal gray matter alterations were negatively correlated with chronic ketamine use (6). In addition, the study observed overall deficits on executive function in chronic ketamine users (6), but the author did not conduct a correlation analysis between gray matter volume changes and executive function. Another study found extensive atrophy in multiple brain regions in patients with 2–4 years of chronic ketamine use, especially in the frontal, parietal, and occipital cortices (7). These findings reflect the deleterious effects of chronic ketamine use on brain regions, such as the PFC (6, 7), which is a key brain region for cognitive functions (e.g., working memory) (8–10). Ketamine use impaired a range of cognitive function, including working memory (11, 12), episodic and semantic memory (11), executive functions (13), and mental and motor speed (13). A previous study found that recognition memory was especially impaired in frequent ketamine users (14). A longitudinal study showed that the impairments caused by ketamine use in spatial working memory and visual recognition were correlated with the level of ketamine use (15). A 3-year longitudinal investigation in ketamine users discovered long-lasting impairments on episodic memory and attentional functioning (12). A previous study found that ketamine use was negatively correlated with performance on spatial working memory and pattern recognition memory (16). But the abnormal brain structure changes that underlie the cognitive deficits in ketamine users have not been elucidated.

Neuroimaging provides an effective methodology to study the abnormal brain structure changes and helps to reveal the underlying neurosubstrate leading to cognitive deficits in ketamine users. Previous VBM study in chronic ketamine users evaluated gray matter volume changes (6), but this evaluation has its limitations. Gray matter volume is more strongly correlated with surface area than cortical thickness. Additionally, VBM cannot separately measure the cortical changes (17), such as cortical thickness. Previous neuroimaging study suggested that surface-based morphometry (SBM) was more sensitive than VBM in detecting gray matter changes (18). SBM independently measures the thickness of the cortex and provides more precise information than VBM on the neuroanatomy changes of brain disorders (17). Cortical thickness reflects the arrangement, size, and density of neurons, neuroglia, and nerve fibers (19). It is sensitive to pathological changes (20) and cognitive function (e.g., executive function) (21, 22). A longitudinal study of cocaine users observed reduced cortical thickness in the lateral frontal regions and the alterations in cortical thickness were related to cognitive performance (attention) changes and cocaine use (23). Nevertheless, to the best of our knowledge, no study has explored cortical thickness abnormalities and its relationship with cognitive function changes in chronic ketamine users. And it is unclear whether the cortical thickness changes in chronic ketamine users are associated with decreased cognitive performance.

In the present study, we use SBM to measure cortical thickness and MATRICS Consensus Cognitive Battery (MCCB) to assess cognitive functions in chronic ketamine users. The purposes of the present study were (1) to perform a comprehensive analysis of the cortical thickness in chronic ketamine users and matched healthy controls (HCs); (2) to investigate the relationship between cortical thickness changes and cognitive performance; (3) to investigate the relationship between cortical thickness changes and clinical and ketamine use characteristics in chronic ketamine users.

## Materials and Methods

### Participants

Chronic ketamine users were enrolled from Guangzhou Huayou Healthcare, Guangdong Provincial Bureau of Drug Rehabilitation, and Guangzhou Baiyun Voluntary Drug Rehabilitation Hospital. HCs were recruited by advertisements. The ethics committee of the Affiliated Brain Hospital of Guangzhou Medical University approved this study. All subjects were given written informed consent before participating in the study. All participants received a semistructured interview to evaluate substance use, sociodemographic characteristics, and psychopathological status.

Here, 28 chronic ketamine users were recruited, whose urine test was positive for ketamine. The inclusion criteria for chronic ketamine users included: (1) patients diagnosed as having ketamine dependence with Diagnostic and Statistical Manual of Mental Disorders, Fourth Edition, Text Revision (DSM-IV-TR); (2) no history of substance dependence (except for ketamine or tobacco); (3) no other substance use (except for tobacco or alcohol) for at least 12 months; and (4) age between 18 and 45 years. Exclusion criteria were: (1) a diagnosis of any substance use disorder (except for ketamine or tobacco) in the past; (2) current or previous psychiatric disorders, antipsychotic treatment, neurological diseases (e.g., serious head injury); (3) intellectual disability; (4) pregnancy; (5) serious medical conditions, such as heart failure; (6) family history of psychiatric disorders (first- or second-degree relatives); (7) major infectious disease (e.g., HIV); (8) positive urine test result for use of other substances, such as methamphetamine, marijuana, cocaine, opioids, and ecstasy.

Thirty HCs were recruited. The inclusion and exclusion criteria were the same as chronic ketamine users (except a history of drug use). The HCs and chronic ketamine users were carefully matched on age, sex, years of education, and race.

Before image scanning, a clinical assessment was performed by psychiatrists. Clinical symptoms were evaluated with the Positive and Negative Syndrome Scale (PANSS) (24, 25) administered by one trained psychiatrist with more than 5 years' clinical experience.

The cognitive function was evaluated by the MCCB (Chinese version) (26, 27), which was translated and revised by Shi and showed good reliability and validity (27). The MCCB consists of 10 tests that measure seven cognitive domains, including verbal learning, visual learning, social cognition, reasoning and problem solving, attention/vigilance, speed of processing, and working memory. The scores of these seven domains are combined to get a composite score (28). Previous studies using MCCB to assess cognitive impairments in schizophrenia (29) and mood disorders (30) have shown that MCCB could provide useful clinical information on the extent of cognitive impairment.

### MRI Data Acquisition

Structural data were acquired using a 3.0-Tesla Philips scanner (Achieva X-series, Philips Medical Systems, the Netherlands) at the Affiliated Brain Hospital of Guangzhou Medical University. A sagittal three-dimensional gradient-echo T1-weighted sequence was performed. The acquisition parameters were repetition time (TR) = 8.2 ms, echo time (TE) = 3.7 ms, flip angle (FA) = 7°, inversion time (TI) = 1,100 ms, acquisition matrix = 256 × 256, field of view (FOV) = 256 mm^2^ × 256 mm^2^, axial slices = 188, slice thickness = 1 mm.

### Data Analysis

Surface-based morphometry was used for the cortical analysis, performed with Computational Anatomy Toolbox (CAT12, http://dbm.neuro.uni-jena.de/cat/). It is an extension with the default pipeline of SPM12 (http://www.fil.ion.ucl.ac.uk/spm/software/spm12/). Cat12 provides a method for automatic measurement of cortical thickness and central surface of hemispheres based on the projection-based thickness (PBT) method (31). Previous study provided evidence that CAT12 makes accurate and robust cortical thickness estimates and can be considered a reliable and fast alternative to FreeSurfer (32). The fully automatic method of CAT12 can access the thickness of the cortex and reconstruct the central surface (31).

All MRI images carry out automatic segmentation of gray matter, white matter, and cerebrospinal fluid, affine registration of MNI template space, and non-linear deformation. A 15-mm FWHM Gaussian kernel was used to smooth resampled surface data of cortical thickness. By using the common second-level models and algorithms provided by the CAT12 software package, we conducted a two-sample *t*-test between the two groups. The cortical thickness of the each hemisphere was compared between groups. Subsequently, we performed region of interest (ROI)-based analysis. The Desikan–Killiany atlas was used to mark the anatomical regions emerging from the between-group comparisons and extracted the ROI-based surface value of the cortical thickness (33). False discovery rate [(FDR)-corrected threshold of *P* < 0.05)] was used to correct for multiple testing.

## Statistical Analyses

Other data statistical analyses were conducted with Statistical Package for Social Sciences version 24 (SPSS, Chicago, USA). We used the Kolmogorov–Smirnov test to investigate the normality of data distribution. Except for age, education, attention/vigilance (AV), and working memory (WM) domains in MCCB, other variables were distributed normally. According to normality of data distribution, the two-sample *t*-test and Mann–Whitney U test were used to compare the differences between the two groups. The α level was set at 0.05. Two-tailed levels of significance (*P* < 0.05) were used. Bonferroni corrections were then used for multiple comparisons in MCCB.

Besides, we were specifically interested in the relationship between clinical data and cortical thickness measures in chronic ketamine users. Pearson correlation (partial correlation) analysis (smoking as covariant) was used to evaluate the correlation between reduced cortical thickness and clinical data, including age of starting ketamine use (year), duration of ketamine use (month), frequency of ketamine use (days per week), average quantity (g) of ketamine use/day, estimated total ketamine consumption, smoking status, and MCCB domains. Benjamini–Hochberg corrections were then applied for reducing the FDR.

## Results

### Demographic Data and Clinical Characteristics of the Participants

Demographic and clinical characteristics of the participants are shown in Table 1. A total of 58 participants (chronic ketamine users/28, HC/30, Chinese Han ethnicity) were recruited in this study. All the participants are male, right-handed. Mean age in the ketamine group was 29.4 years (SD = 5.15), with 11.93 (SD = 2.81) years of education. The chronic ketamine users and HCs were matched for age, educational level, sex, and race. The chronic ketamine users consumed ketamine by snorting the powder. The age of onset of ketamine use was 20.64 ± 4.37 years, and the duration of ketamine use was 107.04 ± 44.24 months. The frequency of ketamine use was 6.018 ± 1.60 (days/week), the average quantity of ketamine use/day was 2.27 ± 1.99 g, and the estimated total ketamine consumption was 3939.6 ± 3873.5 g. There was no frequent alcohol user in either group. Four ketamine users reported occasionally co-use of one or two other drugs, including methamphetamine, ecstasy, gamma-hydroxybutyrate (all polydrug use less than seven occasions). There were no obvious positive and negative symptoms in the ketamine group. Smoking status was available for 14 of the 30 control participants; five were smokers.

**Table 1:** Demographic characteristics of chronic ketamine users and healthy controls (HCs).

**Characteristics**	**Ketamine** **(*n* = 28)**	**Controls** **(*n* = 30)**	***t***	***P***
Age (years)^*^	29.4 ± 5.15	27.8 ± 6.09	−1.69	0.09
Education (years)^*^	11.93 ± 2.81	12.63 ± 2.17	−0.95	0.34
Right-/left-handed	28/0	30/0		
Age at first use of ketamine (years)	20.64 ± 4.37			
Duration of ketamine use (months)	107.04 ± 44.24			
Frequency of ketamine use (days per week)	6.018 ± 1.60			
Average quantity of ketamine use/per day (g)	2.27 ± 1.99			
Estimated total ketamine consumption	3932.1 ± 3830.1			
Frequent alcohol use	0	0		
Other drugs ever used	4			
Methamphetamine	3			
Ecstasy	2			
Gamma-hydroxybutyrate	1			
PANSS	33.89 ± 4.03			
Negative score	7.5 ± 0.92			
Positive score	7 ± 0			
General psychopathology score	19.39 ± 3.68			

* *Mann–Whitney U*.

### Comparison of Cognitive Performance Between Chronic Ketamine Users and Healthy Controls

Compared with the HCs, significant differences between chronic ketamine users and HCs were observed in multiple MCCB domains (Table 2). The chronic ketamine users showed significantly decreased cognitive performance on the working memory (*P* = 0.009), visual learning (*P* = 0.009), speed of processing (*P* < 0.000), and Matrics composite (*P* = 0.01). While there were no significant differences observed on verbal learning, reasoning and problem-solving, social cognition, attention/vigilance domains between the two groups (*P* > 0.05).

**Table 2:** Comparison of cognitive performance of MATRICS consensus cognitive battery (MCCB) between chronic ketamine users and healthy controls (HCs).

**Cognitive domain**	**Ketamine dependents (*N* = 28)**		**Controls(*N* = 30)**		***t***	***P***	***P*_**adjust**_**
	**Mean**	**SD**	**Mean**	**SD**			
VBL	46.29	13.325	46.83	7.202	−0.197	0.845	
VIS	42.71	14.774	51.63	10.029	−2.706	0.009	0.036
RPS	43.64	8.659	47.93	9.044	−1.843	0.071	
SC	51.07	11.122	52.10	9.367	−0.382	0.704	
SOP	37.36	10.867	47.77	8.406	−4.096	0.000	0.001
AV^*^	48.39	7.833	50.33	7.889	−1.037	0.3	
WM^*^	41.79	22.275	44.23	10.271	−2.602	0.009	0.036
Matrics composite	40.96	11.332	47.77	7.934	−1.850	0.01	0.04

* *Mann–Whitney U*.

*VBL, verbal learning; VIS, visual learning; RPS, reasoning and problem-solving speed of processing; SC, social cognition; SOP, speed of processing; AV, attention/vigilance; WM, working memory*.

*P_adjust_: P-value after Bonferroni correction*.

### Group Differences in Regional Cortical Thickness

**Figure 1:**
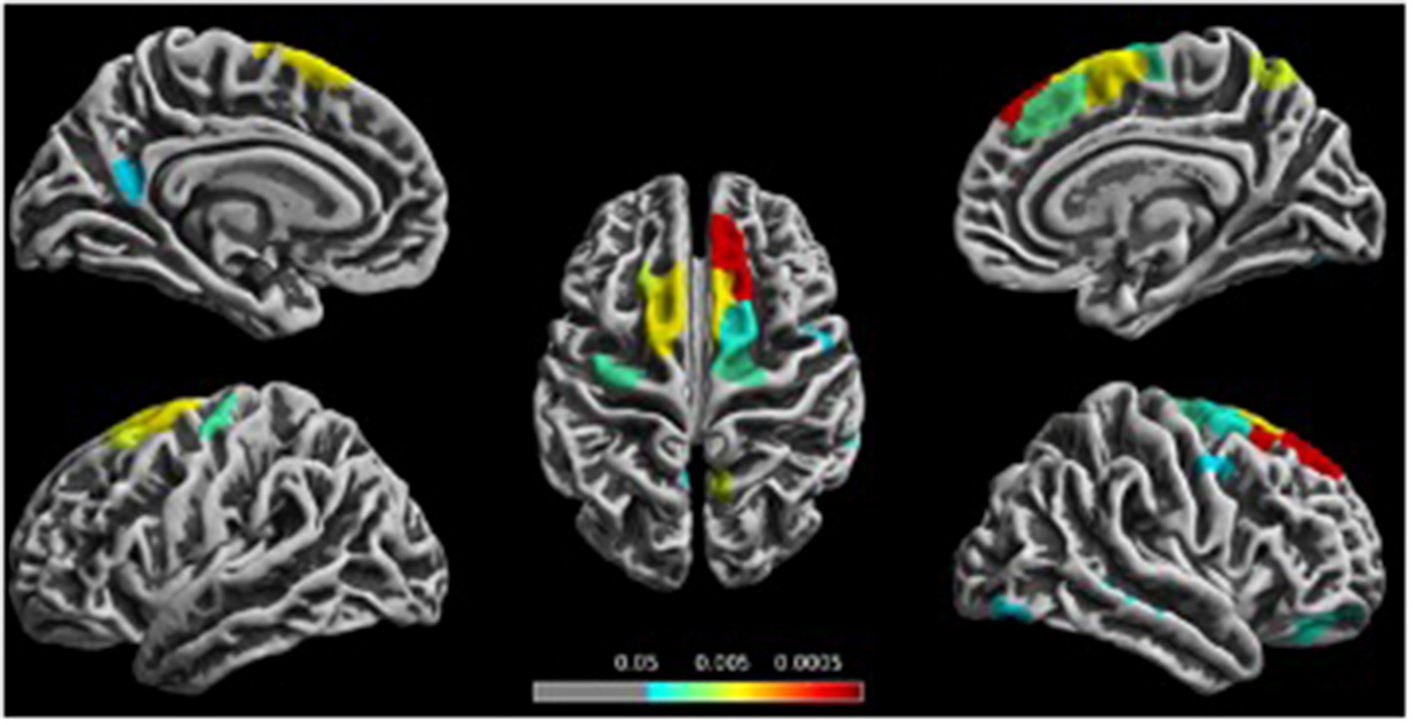
Reduced cortical thickness in the chronic ketamine users compared to the healthy controls (HCs). Figure shows localization of significantly reduced cortical thickness [*P* < 0.05, false discovery rate (FDR)-corrected] in the chronic ketamine users compared to the HC group.

Compared with the HCs, chronic ketamine users exhibited reduced cortical thickness in the bilateral superior frontal gyrus, bilateral precentral gyrus, left isthmus cingulate cortex, right paracentral lobule, right superior parietal, right precuneus, right lateral orbitofrontal, right rostral middle frontal, pars orbitalis, middle temporal, caudal middle frontal, fusiform cortex, and lateral occipital (Figure 1, Table 3).

**Table 3:** Brain regions showing smaller cortical thickness in chronic ketamine users.

**Hemisphere**	**Overlap of atlas region** **(Desikan–Klliany DK40)**	**Vertices**	***P-*value** **(FDR, *P* <0.05)**
**Cortical thickness**			
Left	100% Superior frontal	2,161	0.00321
	100% Precentral	917	0.01679
	65% Precuneus	372	0.04317
	35% Isthmus cingulate		
Right	82% Superior frontal	6,608	0.00026
	14% Precentral		
	4% Paracentral		
	50% Superior parietal	1,061	0.00485
	50% Precuneus		
	80% Lateral orbitofrontal	922	0.02495
	16% Rostral middle frontal		
	4% Pars orbitalis		
	75% Middle temporal	754	0.03322
	25% bankssts		
	67% Precentral	421	0.03943
	33% Caudal middle frontal		
	91% Lateral occipital	364	0.03676
	9% Fusiform		

*P-values refer to false discovery rate (FDR)-corrected values (P < 0.05). Region column shows the label according to the CAT12 output*.

### Relationship Between Clinical Measures, Cognitive Performance, and Reduced Cortical Thickness

As shown in Table 4, the average quantity (g) of ketamine use/day was negatively correlated with cortical thickness in the left superior frontal gyrus (*r* = −0.404, *P* = 0.036), right caudal middle frontal gyrus (MFG) (*r* = −0.386, *P* = 0.047), and right paracentral lobule (*r* = −0.393, *P* = 0.043). The frequency of ketamine use (days per week) was negatively correlated with cortical thickness in the left isthmus cingulate cortex (*r* = −0.49, *P* = 0.01). Duration of ketamine use (month) was negatively correlated with cortical thickness in the left precentral gyrus (*r* = −0.437, *P* = 0.023). However, there was no correlation between any domain of MCCB and cortical thickness, which was significantly reduced in chronic ketamine users (*P* > 0.05). Additionally, we did not find any correlations between cortical thickness changes and age of starting ketamine use, estimated total ketamine consumption.

**Table 4:** Correlation between changes in cortical thickness and clinical associated characteristics in chronic ketamine users.

**Region**	**Age at first use of ketamine**	**Duration of ketamine use** **(months)**	**Frequency of ketamine use**	**Average quantity of ketamine use/per day (g)**	**Estimated total ketamine consumption (g)**
**Center hemisphere**					
Superior frontal	*r* = 0.012	*r* = −0.332	*r* = −0.263	*r* = −0.404	*r* = −0.359
	*P* = 0.951	*P* = 0.090	*P* = 0.185	*P* = 0.036^B^	*P* = 0.066
Precentral	*r* = −0.018	*r* = −0.437	*r* = −0.266	*r* = −0.363	*r* = −0.347
	*P* = 0.931	*P* = 0.023^B^	*P* = 0.181	*P* = 0.063	*P* = 0.076
Precuneus	*r* = 0.010	*r* = −0.402	*r* = −0.309	*r* = −0.348	*r* = −0.332
	*P* = 0.960	*P* = 0.038^B^	*P* = 0.116	*P* = 0.075	*P* = 0.091
Isthmus cingulate	*r* = 0.101	*r* = −0.323	*r* = −0.490	*r* = −0.121	*r* = −0.171
	*P* = 0.617	*P* = 0.100	*P* = 0.010^B^	*P* = 0.549	*P* = 0.393
**Right hemisphere**					
Superior frontal	*r* = −0.084	*r* = −0.311	*r* = −0.155	*r* = −0.366	*r* = −0.315
	*P* = 0.677	*P* = 0.115	*P* = 0.441	*P* = 0.061	*P* = 0.110
Precentral	*r* = −0.152	*r* = −0.335	*r* = −0.053	*r* = −0.294	*r* = −0.250
	*P* = 0.449	*P* = 0.087	*P* = 0.792	*P* = 0.136	*P* = 0.209
Paracentral	*r* = −0.183	*r* = −0.328	*r* = −0.087	*r* = −0.393	*r* = −0.332
	*P* = 0.360	*P* = 0.095	*P* = 0.666	*P* = 0.043^B^	*P* = 0.090
Superior parietal	*r* = −0.102	*r* = −0.177	*r* = −0.024	*r* = −0.329	*r* = −0.240
	*P* = 0.613	*P* = 0.377	*P* = 0.904	*P* = 0.094	*P* = 0.229
Precuneus	*r* = −0.051	*r* = −0.242	*r* = −0.155	*r* = −0.277	*r* = −0.240
	*P* = 0.799	*P* = 0.225	*P* = 0.439	*P* = 0.162	*P* = 0.228
Lateral orbitofrontal	*r* = −0.129	*r* = −0.272	*r* = −0.006	*r* = −0.175	*r* = −0.132
	*P* = 0.521	*P* = 0.170	*P* = 0.975	*P* = 0.382	*P* = 0.512
Rostral middle frontal	*r* = −0.187	*r* = −0.160	*r* = −0.088	*r* = −0.339	*r* = −0.263
	*P* = 0.351	*P* = 0.425	*P* = 0.662	*P* = 0.084	*P* = 0.185
Pars orbitalis	*r* = −0.069	*r* = 0.031	*r* = 0.112	*r* = −0.236	*r* = −0.088
	*P* = 0.732	*P* = 0.878	*P* = 0.578	*P* = 0.236	*P* = 0.664
Middle temporal	*r* = −0.262	*r* = −0.216	*r* = −0.132	*r* = −0.274	*r* = −0.185
	*P* = 0.186	*P* = 0.278	*P* = 0.511	*P* = 0.167	*P* = 0.354
Caudal middle frontal	*r* = −0.183	*r* = −0.220	*r* = −0.046	*r* = −0.386	*r* = −0.344
	*P* = 0.360	*P* = 0.270	*P* = 0.821	*P* = 0.047^B^	*P* = 0.079
Lateral occipital	*r* = −0.129	*r* = −0.208	*r* = −0.074	*r* = −0.257	*r* = −0.234
	*P* = 0.522	*P* = 0.299	*P* = 0.713	*P* = 0.196	*P* = 0.241
Fusiform	*r* = −0.177	*r* = −0.107	*r* = −0.024	*r* = −0.139	*r* = −0.167
	*P* = 0.377	*P* = 0.594	*P* = 0.904	*P* = 0.489	*P* = 0.406

*Correlations that survived Benjamini–Hochberg correction are marked by the letter “B”*.

## Discussion

In the present study, we found that cortical thickness was significantly reduced in the frontal, parietal, temporal, and occipital lobes in chronic ketamine users compared to HCs. We also observed negative correlations between the cortical thickness and dosage, duration, and frequency of ketamine use. But no correlation between cortical thickness and scores of domain of MCCB was found.

To the best of our knowledge, our study is the first to analyze the cortical thickness in chronic ketamine users. The present study found reduced cortical thickness in the left superior frontal gyrus, right caudal middle frontal gyrus (MFG). A previous neuroimaging study of chronic ketamine users observed a significant decrease in the gray matter volume of the left superior frontal gyrus and right middle frontal gyrus, and the reduced gray matter volume was correlated with the duration of ketamine use and estimated total ketamine consumption (6). In agreement with this study, we found reduced cortical thickness in similar brain regions in chronic ketamine users. Besides, the present study also found reduced cortical thickness in bilateral frontal cortices and parietal, temporal, and occipital lobes of chronic ketamine users. Our results indicated more abnormal gray matter changes in chronic ketamine users.

A previous study indicated that the neurotoxic effects of ketamine could cause neuronal apoptosis (34–36). Administration of ketamine 20 mg/kg resulted in a significant increase in neuronal death in rats (35). Ketamine infusion for a prolonged duration caused significant neuronal damage in the frontal cortex of rhesus monkeys (37, 38). The neurotoxic effects of ketamine depended on the frequency, dosage, and duration of ketamine administration (39). Consistent with a previous study, the present study found that the frequency, duration, and average quantity of ketamine used were correlated with abnormal cortical thickness changes, which was most prominent in the frontal lobes of chronic ketamine users (frontal cortices and paracentral lobule). These reduced cortical thicknesses are probably related to neuronal cell death due to neurotoxicity of ketamine. Therefore, it is crucial to interfere with the use of ketamine in the early stage to prevent the neurotoxic effect of ketamine on the brain.

The present study found reduced cortical thickness in the left superior frontal gyrus, MFG, and right paracentral lobule. These brain regions are related to cognitive control (40–43), which was highly involved in goal-directed behavior, decision-making, inhibition of impulses, and inhibition of conditioned behavior (41, 44, 45). The reduced cortical thickness of the PFC was correlated with the impaired cognitive control (46, 47). The deficits in cognitive control is a critical factor leading to continued drug use in individuals with addictive disorders (48–51). The present study observed multiple gray matter atrophy in the brain regions of cognitive control network. Therefore, as the neural structure supporting cognitive control deteriorated, the function of cognitive control was probably weakened, and the chronic ketamine users may have difficulty in controlling drug use. On the other hand, the reduced cortical thickness in bilateral PFC and paracentral lobule may lead to a higher dose of ketamine use due to decreased function of cognitive control. Meanwhile, the current study found reduced cortical thickness in the left isthmus cingulate cortex [part of the anterior cingulate (ACC)], and the cortical thickness in the left isthmus cingulate cortex (part of the ACC) was negatively correlated with the frequency of ketamine use in chronic ketamine users. The ACC was related to response inhibition (52). A previous study (53) suggested that the response inhibition is crucial to restrict drug cravings and drug-seeking behaviors. Also, response inhibition is a central construct for abstinence, and deficits in response inhibition characterize substance dependence. The cortical thickness in the ACC was associated with response control (47). The reduced cortical thickness in the left isthmus cingulate may reflect decreased response inhibition (54) and increased drug-seeking behaviors (55). Reduced cortical thickness in the left isthmus cingulate cortex may ultimately result in frequent use of ketamine in chronic ketamine users. On the other hand, due to the neurotoxicity of ketamine, the abnormal cortical changes found in the brain regions discussed above might be at least partially induced by chronic ketamine use.

We also found reduced cortical thickness in the left precentral gyrus, and a negative correlation between cortical thickness in the left precentral gyrus and duration of ketamine use was observed in chronic ketamine users. Previous studies of brain structure in methamphetamine (56, 57), marijuana (58), and alcohol users (59) have also reported reduced gray matter volume in the precentral gyrus. Duration of methamphetamine use was positively associated with a reduction in gray matter in the left precentral gyrus (56). Considering the neurotoxic effect of ketamine, we speculate that the reduced cortical thickness of the left precentral gyrus in chronic ketamine users may be a consequence of long-term drug consumption.

In summary, our study found abnormal cortical alterations in the prefrontal cortex (PFC), which was consistent with a previous study (6). But we found more abnormal cortical alterations in multiple brain regions, and the correlations between chronic ketamine use and abnormal gray matter changes are different from those of a previous study (6). These inconsistencies could be potentially attributed to the different methods of measuring gray matter changes. A previous study based on VBM used an ROI approach to analyze the gray matter volume changes in frontal cortices, while the current study based on SBM performed whole-brain vertex-level cortical thickness analysis. Evidence indicated that the SBM method is more sensitive than VBM in detecting gray matter changes, and cortical thickness analysis detects more changes in the gray matter layer compared with VBM (18). Besides, the assessment of gray matter volume by VBM reflects combined information that includes surface area, cortical folding, and cortical thickness (17, 60), therefore less specific. While the SBM method used in the current study reflected specific information of cortical thickness. The meaning of cortical thickness changes is not completely consistent with that of gray matter volume, which may lead to different correlational findings between abnormal gray matter changes and chronic ketamine use.

The present study also found reduced cortical thickness in orbitofrontal, precuneus, lateral occipital, parietal, and fusiform. These abnormal gray matter changes might relate to chronic ketamine use, but we did not observe any correlation between chronic ketamine use and cortical thickness in these brain regions. The consequences of these cortical changes and their relationship with ketamine use need to be further explored in future studies.

Consistent with previous researches (11, 13, 16, 61–63), the current study found deficits on cognitive domains of MCCB in chronic ketamine users, including working memory, visual learning, speed of processing, Matrics composite. Working memory, visual learning, and speed of processing were associated with the PFC (61, 64, 65). But the present study did not find correlations between cortical thickness changes and decreased cognitive performance. The domains of cognition, such as working memory (64) and speed of processing (66), are usually associated with a range of brain regions. It was possible that the functional impairments of specific brain regions could be compensated by itself or other brain regions (67). These neural compensatory mechanisms might postpone the onset of cognitive impairments. Besides, there may be individual differences in the mechanism of neural compensation. In view of this, the abnormal structure changes might not be plainly correlated with decreased cognitive performance. Also, our sample size is relatively small; there may not be adequate statistical power to detect the correlation between cortical thickness changes and cognitive performance in the current study. Further studies with larger sample sizes are needed to clarify the relationship between abnormal cortical thickness changes and cognitive performance in chronic ketamine users.

Also, ketamine is widely used as a pharmacological model of schizophrenia to mimic the psychotic symptoms and cognitive deficits (68). The psychotic symptom dimensions in chronic ketamine users were similar to those observed in schizophrenia patients (69). The schizophrenia patients showed broad reduced gray matter volume and cortical thickness in multiple brain regions (70–72), such as the superior frontal gyrus, middle temporal gyrus, and isthmus cingulate. Although acute ketamine administration cannot induce the abnormal gray matter changes similar to those observed in schizophrenia patients (73), chronic ketamine use could induce reduced gray matter volume or cortical thickness in bilateral frontal, parietal, temporal, and occipital lobes, which is very similar to those found in schizophrenia (70–72). Furthermore, a previous study in chronic ketamine users found decreased white matter fractional anisotropy in the medial frontal, which are remarkably in accordance with those shown in schizophrenia (74). In terms of chronic illness course, pattern of psychotic symptoms, cognitive deficits, and abnormalities in brain structure, chronic ketamine use may provide a better model of schizophrenia.

### Limitation

Findings from the current study should be interpreted with several limitations in mind. First, the sample size was modest; nevertheless, our findings were primarily consistent with a previous study using different methods and analytic approaches (6), supporting the validity of our results. Second, as a cross-sectional study, the causal relationship between abnormal cortical alterations and chronic ketamine use could not be concluded. Preexisting abnormal gray matter changes may also predispose individuals to engage in substance use (75, 76). We are prone to consider that the abnormal cortical thickness changes in chronic ketamine users were at least partially induced by ketamine use. Longitudinal investigations will be important for a clear and definite understanding. Third, our study did not match tobacco use between the two groups, but we did not find any correlation between reduced cortical thickness and estimated total smoked cigarettes (*P* > 0.12). In line with the current study, previous neuroimaging study of chronic ketamine users did not find any correlation between reduced gray matter volume and estimated total smoked cigarettes (6). Although our analyses suggested that the smaller cortical thickness we observed is unlikely to reflect group differences in smoking, we cannot exclude the potential effect of smoking on cortical thickness, which should be taken into account in interpreting our results. Future studies should match groups on smoking status and history. Fourth, although we try to include only subjects who are rarely used in combination with other substances, exposure to other substances is almost inevitable for most drug addicts. In the present study, only four patients reported a history of co-use substance (all polydrug use of <7 lifetime occasions), and we can almost dismiss the influence of other substances. Finally, female ketamine users were not included in this study, so we cannot determine whether the present findings generalize to female ketamine users.

## Conclusion

The present study showed reduced cortical thickness in the frontal, parietal, temporal, and occipital lobes in chronic ketamine users, especially in frontal cortices. Our results indicated that the dose, frequency, and duration of ketamine use were correlated to abnormal cortical thickness changes in chronic ketamine users. But the present study did not observe any correlation between reduced cortical thickness and decreased cognitive performance in chronic ketamine users. Longitudinal studies will be very important in the future. It can help us find out whether these abnormal changes in the gray matter of chronic ketamine users can be recovered or be used to predict prognosis.

## Data Availability Statement

The raw data supporting the conclusions of this article will be made available by the authors, without undue reservation.

## Ethics Statement

The studies involving human participants were reviewed and approved by The ethics committee of the Affiliated Brain Hospital of Guangzhou Medical University. The patients/participants provided their written informed consent to participate in this study.

## Author Contributions

NF and HH designed the study and wrote the protocol. JZ and ZZ recruited the subjects and undertook the statistical analysis. NF and JZ wrote the manuscript. HW, PC, and JH contributed to data input. JZ and FW helped enrolling volunteers. All authors contributed to and have approved the final manuscript.

## Conflict of Interest

The authors declare that the research was conducted in the absence of any commercial or financial relationships that could be construed as a potential conflict of interest.
